# Supplementary material for: We’re all in this together: Focus on community attenuates effects of pandemic-related financial hardship on reactance to COVID-19 public health regulations
Source: PLoS One. 2021 Dec 23;16(12):e0260782. doi: 10.1371/journal.pone.0260782 (PMC8699617; doi:10.1371/journal.pone.0260782)
Supplement: Attachment — Submitted filename: Response to Reviewers.docx [file pone.0260782.s002.docx]

We thank the anonymous Reviewer for the thorough and helpful reviews they provided to the original manuscript. We have endeavored to respond to each of the comments raised in the revised manuscript and in this response letter. The specific ways in which we have responded to each of the comments is detailed below *with our responses provided in italicized font.*
Reviewers' comments:

6. Review Comments to the Author

Reviewer #2: The authors sufficiently responded to the comments from the Editor and Reviewer. I appreciate the amount of detail and effort that went into each of the responses. In particular, I appreciate the inclusion of gender in the analyses. I also appreciate the inclusion of subheadings in the introduction, as this makes for an easier read. MAJOR NOTES

1. Following up on the original comment from Reviewer 1, the replicability link still does not work, showing that the depository is empty.

*We are able to view and download the data and associated analytic script when we click the replicability link, and we are unsure why the depository is empty for both you and the Editor. Since receiving the revision request, we have deleted and reuploaded the files, and had multiple lab members check and they were able to view and download the files. This should be remedied and allow for a complete review.*

2. Where did the item for “Adherence to Social Distancing” come from? Is this pulled from another questionnaire, or was it developed by the authors themselves for the purpose of this study? Please state either way.

*We created this item for the purposes of the study and have clarified this in the “Adherence to Social Distancing” subsection (p. 12).*

3. Was the wording of the 6 items from Conger and colleagues’ economic hardship measures adapted to include “since the pandemic began”? If so, please state that you adapted/changed this measure to meet the needs of the current study.

*We have added the following sentence to specify that the wording was changed for these measures in order to better capture financial stress in association with COVID-19, “The prompt “Since the pandemic began” was added to items to orient participants to rating financial stress experienced in the wake of the pandemic” (p. 12).*

4. Under “Psychological Sense of Community”, you provide an example item for the two items measuring personal identification, but you do not provide an example item for the two items measuring connection. While not a huge deal, I recommend also including an example item of connection for your readers.

*We appreciate the recommendation and have added the following example item of connection: “Two items measured participants’ connection to those impacted by COVID-19 (e.g., “I feel strong ties to the community affected by COVID-19”) and two additional items measured participants’ personal identification with those impacted by COVID-19 (e.g., “I identify with the community affected by COVID-19”)” (p. 13-14).*

5. You don’t mention income as a covariate in your introduction, but you mention it in your method section, and even use it as an exclusion criterion if not answered. Perhaps briefly mention it in your “Current Study” section of the introduction.

*Thank you for this suggestion; we now identify pre-pandemic income as a covariate in the Current Study section (p. 9).*

6. Table 1 is very helpful, and I appreciate its inclusion in the manuscript. However, it does not follow APA formatting guidelines. Additionally, many of the values do not line up, such that some are flush left, and some are centered, making it harder to read. Please reformat this table (e.g., remove all vertical lines and any excess horizontal lines, center all values, etc.).

*Table 1 has been reformatted to meet APA 7 and PLOS One guidelines for Tables. All values are centered, except the first column containing variables names which left-centered for ease of reading associated with long text entries (e.g., names, etc.). Additionally, all unnecessary horizontal and vertical lines have been removed.*

7. You mention that those in the community-focused condition were more politically conservative (M = 2.99, SD = 1.46) compared to those in the self-focused condition, M = 2.57, SD = 1.53, t(299) = -2.44, p = .015, 95% CI: [-0.76, -0.08]. Do you think this could have impacted the lack of effect that restriction condition had on reactance, given that political orientation significantly impacted reactance? It is possible I missed where this was discussed or ruled out in the analyses. But if not, please make mention of this either in your analyses or briefly in your discussion.

*We understand the Reviewer’s concerns and have added an Exploratory Post Hoc Analysis subsection to the Results (pp. 20-21) to address the possibility of a restriction condition x political orientation interaction impacting results (p. 20-21). Of note, including this interaction resulted in poor model fit,* $\chi^{2}$*(30) = 274.31, p < .001, robust CFI = .74, robust TLI =.61, robust RMSEA = .19 (90% CI: [.17, .21), and the restriction condition x political orientation interaction was nonsignificant, b = -0.01, p = .85. Moreover, the main effect of restriction condition remained nonsignificant, b = .39, p = .09, while the significant restriction condition x financial stress interaction remained significant, b = -0.29, p = .03. Finally, we have added the following sentence to the Limitations section of the Discussion to acknowledge the possibility of a more politically conservative community-focused group may have influenced the restriction condition main effect: “Despite random assignment to restriction condition, the disproportionate representation of more politically conservative participants within the community-focused group may have elevated reactance within this condition and reduced the magnitude of the predicted difference between restriction conditions;* *future research should explore this possibility further” (p. 26). Our hope is that these additions to the manuscript better address the potential impact of political orientation on our results.*

8. Why is gender listed in Fig. 3 but not Fig. 2?
9. The disproportionate distribution of gender was not mentioned in your limitations section. I recommend mentioning this alongside the statement about ethnicity.

*We have added a paragraph to the Strengths and Limitations subsection that discusses the disproportionate distribution of gender as a limitation and that contextualizes our findings within the gender and COVID-19 literature (p. 26).*

MINOR NOTES

1. Throughout the introduction, the United States is sometimes written as U.S. and other times is written as US. Please keep consistent throughout.

*We have corrected the error and the United States should now be written as U.S. throughout the manuscript.*

2. For figures 1a and 1b: in the text, the a and b are lowercase but, in the figures, they are uppercase (A; B). Please keep consistent.

*We have corrected the error and have capitalized A and B within the text to maintain consistency with the figure.*

3. In some places, you round to three decimals, and in other places, you round to two. For example, the Cronbach’s alpha for State Reactance was .896, 95% CI [.867, .917]. I recommend keeping it to two decimals throughout.

*We have corrected the error and have all decimals rounded to two decimal places, except for p-values.*

4. Under the “State Reactance” subheading, please change the following sentence from “The scale consisted of 11items assessing experience of reactance” to “The scale consisted of 11 items assessing experience of reactance” (insert space between 11 and items).

*We have corrected the typological error and have inserted a space between “11” and “items” for this sentence.*

5. You included gender in your Fig. 3 model, but it is not named in the caption for Fig. 3. Please include alongside mention of the other variables.

*We have corrected our error and have listed gender in the figure caption.*
